# Supplementary material for: Resistance mechanisms of cereal plants and rhizosphere soil microbial communities to chromium stress
Source: PeerJ. 2024 Jun 28;12:e17461. doi: 10.7717/peerj.17461 (PMC11216213; doi:10.7717/peerj.17461)
Supplement: Supplemental Information 1 [file peerj-12-17461-s001.docx]

| Target gene | Primer | Function |
| --- | --- | --- |
| AOA*-amoA* | CrenamoA23F (ATGGTCTGGCTWAGACG)  CrenamoA 616R (GCCATCCATCTGTATGTCCA) | Nitrification |
| AOB*-amoA* | Bac-amoA-1F (GGGGTTTCTACTGGTGGT)  Bac-amoA- 2R (CCCCTCKGSAAAGCCTTCTTC) | Nitrification |
| *narG* | NifHF (TCGCCSATYCCGGCSATGTC)  NifHRb (GAGTTGTACCAGTCRGCSGAYT CSG) | Denitrification |
| *nirK* | nirK876 (ATYGGCGGVCAYGGCGA)  nirK1040(GCCTCGATCAGRTTRTGGTT) | Denitrification |
| *nifH* | nifH F (GGTGGTGTMGGATTCACACARTAYGCWACAGC)  nifH R (TTCATTGCRTAGTTWGGRTAGTT) | Ammonification |
| *pmoA* | A189F_ (GGNGACTGGGACTTCTGG)  mb661R (CCGGMGCAACGTCYTTACC) | Methane generation |
| *mcrA* | mcrA-F_ (GGTGGTGTMGGATTCACACARTAYGCWACAGC)  mcrA-R (TTCATTGCRTAGTTWGGRTAGTT) | Methane oxidation |
