# Supplementary material for: Resistance mechanisms of cereal plants and rhizosphere soil microbial communities to chromium stress
Source: PeerJ. 2024 Jun 28;12:e17461. doi: 10.7717/peerj.17461 (PMC11216213; doi:10.7717/peerj.17461)
Supplement: Supplemental Information 2 — Raw reads: total number of entries of original sequencing data; Clean reads: total number of items of sequencing data after quality control; Q20 (%), Q30 (%): refers to the percentage of bases with sequencing quality above 99% and 99.9% in the total bases, respectively; GC content (%): percentage of the total G and C bases corresponding to the quality control data in the total bases. [file peerj-12-17461-s002.docx]

| Sample | Raw reads | Clean reads | Q20 (%) | Q30 (%) | GC content (%) |
| --- | --- | --- | --- | --- | --- |
| CK_1 | 43160218 | 42741044 | 98.41 | 95.11 | 54.99 |
| CK_2 | 42282286 | 41694490 | 98.27 | 94.82 | 55.18 |
| CK_3 | 42848634 | 42277974 | 98.26 | 94.78 | 55.19 |
| Cr6h_1 | 44553716 | 43912092 | 98.39 | 95.08 | 54.35 |
| Cr6h_2 | 40447840 | 39771594 | 98.22 | 94.7 | 54.57 |
| Cr6h_3 | 44176666 | 43493968 | 98.42 | 95.2 | 53.83 |
| Cr6d_1 | 43345554 | 42906542 | 98.28 | 94.83 | 54.46 |
| Cr6d_2 | 47011994 | 46473272 | 98.43 | 95.2 | 54.34 |
| Cr6d_3 | 45313342 | 44913500 | 98.43 | 95.22 | 54.79 |
