# Supplementary material for: Resistance mechanisms of cereal plants and rhizosphere soil microbial communities to chromium stress
Source: PeerJ. 2024 Jun 28;12:e17461. doi: 10.7717/peerj.17461 (PMC11216213; doi:10.7717/peerj.17461)
Supplement: Supplemental Information 3 [file peerj-12-17461-s003.docx]

| Functional Categories | CK&Cr_6h | | CK&Cr_6d | | Cr_6h&Cr_6d | |
| --- | --- | --- | --- | --- | --- | --- |
|  | up | down | up | down | up | down |
| Function unknown | 1569 | 874 | 711 | 603 | 1190 | 1658 |
| Posttranslational modification, protein turnover, chaperones | 113 | 101 | 50 | 57 | 115 | 103 |
| Transcription | 105 | 65 | 66 | 28 | 99 | 110 |
| Carbohydrate transport and metabolism | 123 | 50 | 42 | 43 | 66 | 115 |
| Signal transduction mechanisms | 83 | 48 | 61 | 25 | 94 | 64 |
| Translation, ribosomal structure and biogenesis | 82 | 23 | 34 | 14 | 18 | 55 |
| Amino acid transport and metabolism | 45 | 35 | 25 | 16 | 55 | 40 |
| Secondary metabolites biosynthesis, transport and catabolism | 54 | 21 | 30 | 12 | 32 | 43 |
| Intracellular trafficking, secretion, and vesicular transport | 47 | 27 | 18 | 18 | 34 | 38 |
| Energy production and conversion | 26 | 29 | 15 | 37 | 29 | 16 |
| Replication, recombination and repair | 48 | 13 | 20 | 9 | 19 | 24 |
| Lipid transport and metabolism | 21 | 19 | 15 | 10 | 32 | 29 |
| Cell wall/membrane/envelope biogenesis | 28 | 11 | 15 | 6 | 26 | 28 |
| Inorganic ion transport and metabolism | 12 | 26 | 8 | 11 | 32 | 14 |
| Coenzyme transport and metabolism | 19 | 8 | 10 | 7 | 11 | 23 |
| Defense mechanisms | 17 | 11 | 9 | 4 | 15 | 13 |
| Cell cycle control, cell division, chromosome partitioning | 20 | 8 | 12 | 6 | 7 | 15 |
| Nucleotide transport and metabolism | 20 | 4 | 13 | 3 | 7 | 11 |
| Chromatin structure and dynamics | 19 | 0 | 4 | 3 | 4 | 14 |
| Cytoskeleton | 11 | 6 | 3 | 4 | 2 | 10 |
| RNA processing and modification | 7 | 6 | 2 | 2 | 6 | 8 |
