# Supplementary material for: Resistance mechanisms of cereal plants and rhizosphere soil microbial communities to chromium stress
Source: PeerJ. 2024 Jun 28;12:e17461. doi: 10.7717/peerj.17461 (PMC11216213; doi:10.7717/peerj.17461)
Supplement: Supplemental Information 4 [file peerj-12-17461-s004.docx]

**Table S4.** GO enrichment analysis in STEM

| Profiles | GO term | Go descriptipn | No. of DEGs | p-value | p-adjust |
| --- | --- | --- | --- | --- | --- |
| 10 | BP | intercellular transport | 12 | 2.84768E-07 | 0.000350975 |
|  |  | plasmodesmata-mediated intercellular transport | 12 | 2.84768E-07 | 0.000350975 |
|  |  | ribosomal large subunit assembly | 22 | 4.37788E-07 | 0.000350975 |
|  |  | chloroplast fission | 15 | 4.46131E-07 | 0.000350975 |
|  |  | plastid fission | 15 | 4.46131E-07 | 0.000350975 |
|  |  | plastid transcription | 8 | 1.05163E-06 | 0.000350975 |
|  |  | plant-type secondary cell wall biogenesis | 26 | 1.1561E-06 | 0.000350975 |
|  |  | microtubule-based movement | 32 | 1.24068E-06 | 0.000350975 |
|  |  | chloroplast organization | 60 | 1.28686E-06 | 0.000350975 |
|  |  | establishment of protein localization to membrane | 36 | 1.3162E-06 | 0.000350975 |
|  |  | movement of cell or subcellular component | 39 | 1.4446E-06 | 0.000350975 |
|  |  | cytoskeleton organization | 51 | 1.79843E-06 | 0.000350975 |
|  |  | protein targeting | 53 | 1.83324E-06 | 0.000350975 |
|  |  | plastid organization | 68 | 1.83937E-06 | 0.000350975 |
|  |  | microtubule-based process | 69 | 2.04833E-06 | 0.000350975 |
|  |  | protein localization to chloroplast | 17 | 2.14261E-06 | 0.000350975 |
|  |  | cytoplasmic translation | 19 | 2.30809E-06 | 0.000350975 |
|  | CC | chloroplast nucleoid | 15 | 2.90628E-07 | 0.000350975 |
|  |  | endopeptidase Clp complex | 10 | 3.09723E-07 | 0.000350975 |
|  |  | mitochondrial respiratory chain complex I | 23 | 4.27882E-07 | 0.000350975 |
| 14 | BP | rRNA pseudouridine synthesis | 7 | 4.71999E-08 | 9.0379E-05 |
|  |  | snoRNA 3'-end processing | 9 | 6.49041E-08 | 9.0379E-05 |
|  |  | nuclear RNA surveillance | 8 | 1.61372E-07 | 0.000140443 |
|  |  | snoRNA metabolic process | 9 | 1.93967E-07 | 0.000140443 |
|  |  | snoRNA processing | 9 | 1.93967E-07 | 0.000140443 |
|  |  | maturation of SSU-rRNA from tricistronic rRNA transcript | 13 | 2.50158E-07 | 0.000140443 |
|  |  | snRNA metabolic process | 14 | 2.63059E-07 | 0.000140443 |
|  |  | pseudouridine synthesis | 14 | 3.43861E-07 | 0.000142201 |
|  |  | regulation of helicase activity | 7 | 3.47963E-07 | 0.000142201 |
|  |  | U4 snRNA 3'-end processing | 7 | 3.47963E-07 | 0.000142201 |
|  |  | mitochondrial transport | 24 | 4.15098E-07 | 0.000142201 |
|  | CC | cytoplasmic exosome (RNase complex) | 8 | 3.50662E-08 | 9.0379E-05 |
|  |  | box H/ACA RNP complex | 7 | 4.71999E-08 | 9.0379E-05 |
|  |  | box H/ACA snoRNP complex | 7 | 4.71999E-08 | 9.0379E-05 |
|  |  | small-subunit processome | 18 | 2.14469E-07 | 0.000140443 |
|  |  | exosome (RNase complex) | 13 | 2.373E-07 | 0.000140443 |
|  |  | U2 snRNP | 16 | 2.63059E-07 | 0.000140443 |
|  |  | mitochondrial large ribosomal subunit | 20 | 2.68951E-07 | 0.000140443 |
|  |  | organellar large ribosomal subunit | 20 | 2.68951E-07 | 0.000140443 |
| 11 | BP | ribonucleoprotein complex biogenesis | 15 | 3.32323E-07 | 0.000476491 |
|  |  | tRNA modification | 18 | 3.66522E-07 | 0.000476491 |
|  |  | rRNA processing | 29 | 3.93732E-07 | 0.000476491 |
|  |  | macromolecule methylation | 23 | 4.23801E-07 | 0.000476491 |
|  |  | RNA methylation | 15 | 4.51779E-07 | 0.000476491 |
|  |  | tRNA processing | 24 | 4.97006E-07 | 0.000476491 |
|  |  | tRNA methylation | 10 | 6.14679E-07 | 0.000476491 |
|  |  | rRNA metabolic process | 30 | 7.60858E-07 | 0.000476491 |
|  |  | peptidyl-amino acid modification | 34 | 8.40208E-07 | 0.000476491 |
|  |  | tRNA metabolic process | 31 | 1.00641E-06 | 0.000476491 |
|  |  | nucleocytoplasmic transport | 18 | 1.0751E-06 | 0.000476491 |
|  |  | nuclear transport | 18 | 1.0751E-06 | 0.000476491 |
|  |  | intracellular protein transport | 52 | 1.10185E-06 | 0.000476491 |
|  |  | establishment of protein localization | 61 | 1.1652E-06 | 0.000476491 |
|  |  | ncRNA metabolic process | 63 | 1.22295E-06 | 0.000476491 |
|  |  | maturation of 5.8S rRNA | 7 | 1.33012E-06 | 0.000476491 |
|  |  | amide transport | 62 | 1.33041E-06 | 0.000476491 |
|  |  | establishment of localization in cell | 65 | 1.3916E-06 | 0.000476491 |
|  |  | intracellular transport | 63 | 1.40811E-06 | 0.000476491 |
|  |  | macromolecule localization | 64 | 1.49084E-06 | 0.000476491 |
| 15 | BP | RNA processing | 36 | 2.04432E-06 | 0.005323768 |
|  |  | nucleobase-containing compound metabolic process | 91 | 2.83612E-06 | 0.005323768 |
|  |  | RNA metabolic process | 55 | 3.07163E-06 | 0.005323768 |
|  |  | heterocycle metabolic process | 93 | 3.45397E-06 | 0.005323768 |
|  |  | cellular aromatic compound metabolic process | 94 | 3.83831E-06 | 0.005323768 |
|  |  | cellular process | 304 | 3.88564E-06 | 0.005323768 |
|  |  | nucleic acid metabolic process | 73 | 4.46037E-06 | 0.005323768 |
|  |  | organic cyclic compound metabolic process | 96 | 5.72579E-06 | 0.005979876 |
|  |  | carbohydrate derivative metabolic process | 27 | 1.77827E-05 | 0.015067893 |
|  |  | cellular nitrogen compound metabolic process | 100 | 1.80346E-05 | 0.015067893 |
|  |  | pentose-phosphate shunt | 5 | 2.41212E-05 | 0.016837713 |
|  |  | NADP metabolic process | 6 | 2.41834E-05 | 0.016837713 |
|  |  | NADPH regeneration | 5 | 3.16537E-05 | 0.020096014 |
|  |  | intracellular transport | 29 | 3.67948E-05 | 0.020096014 |
|  |  | biological_process | 378 | 3.8681E-05 | 0.020096014 |
|  |  | glucose 6-phosphate metabolic process | 5 | 4.08896E-05 | 0.020096014 |
|  |  | tRNA modification | 9 | 5.0302E-05 | 0.021313048 |
|  | MF | omega peptidase activity | 10 | 3.91642E-05 | 0.020096014 |
|  | CC | cytoplasmic vesicle | 15 | 4.85763E-05 | 0.021313048 |
|  |  | intracellular vesicle | 15 | 5.10187E-05 | 0.021313048 |
