# Supplementary figures and images for: Resistance mechanisms of cereal plants and rhizosphere soil microbial communities to chromium stress
Source: PeerJ. 2024 Jun 28;12:e17461. doi: 10.7717/peerj.17461 (PMC11216213; doi:10.7717/peerj.17461)

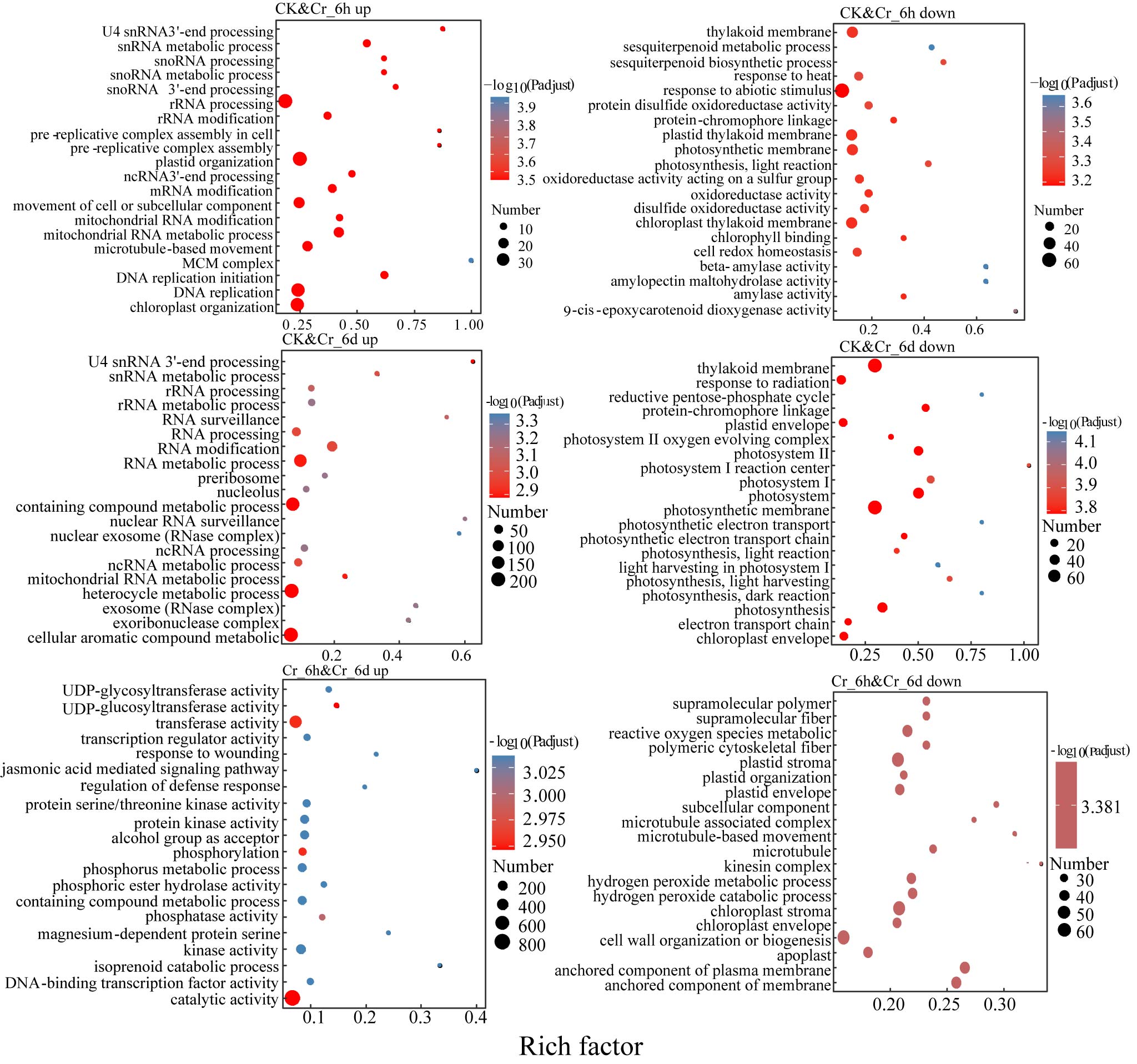

Supplement: Supplemental Information 5 [file peerj-12-17461-s005.jpg]

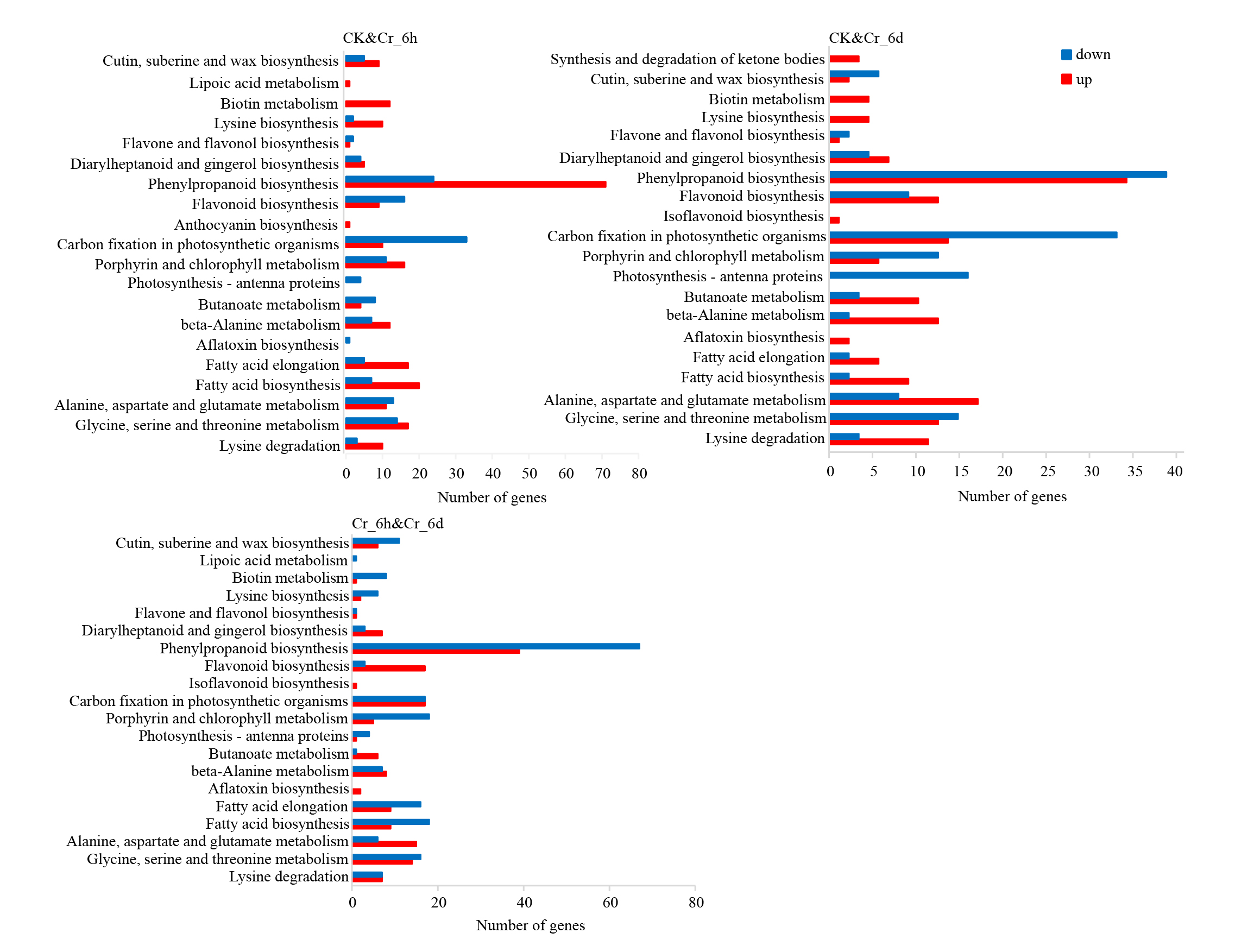

Supplement: Supplemental Information 6 [file peerj-12-17461-s006.jpg]
