# Supplementary material for: Resistance mechanisms of cereal plants and rhizosphere soil microbial communities to chromium stress
Source: PeerJ. 2024 Jun 28;12:e17461. doi: 10.7717/peerj.17461 (PMC11216213; doi:10.7717/peerj.17461)
Supplement: Supplemental Information 7 [file peerj-12-17461-s007.docx]

| Abbreviation | Full name |
| --- | --- |
| BP | Biological process |
| CC | Cellular component |
| Cr | Chromium |
| Cr_6d | Cr stress for 6 days |
| Cr_6h | Cr stress for 6 hours |
| DEGs | Differentially expressed genes |
| GO | Gene ontology |
| HMs | Heavy metals |
| KEGG | Kyoto encyclopedia of genes and genomes |
| MF | Molecular function |
